# Investigating the eukaryotic host-like SLiMs in microbial mimitopes and their potential as novel drug targets for treating autoimmune diseases

**DOI:** 10.3389/fmicb.2022.1039188

**Published:** 2022-11-04

**Authors:** Anjali Garg, Neelja Singhal, Manish Kumar

**Affiliations:** Department of Biophysics, University of Delhi South Campus, New Delhi, India

**Keywords:** motif mimicry, short linear motifs (SLiMs), autoimmunity, mimitopes, drug targets

## Introduction

The host genetics play an important role in the induction of autoimmune response against self-antigens however; several epidemiological and molecular evidences suggest microbial pathogens (viruses and bacteria) are the principal environmental triggers of autoimmunity (Fujinami, 2001; Ascherio and Munger, 2007; Libbey and Fujinami, 2010; Tarakhovsky and Prinjha, 2018; Lester et al., 2019). Reportedly, four main criteria have been associated with molecular mimicry and the underlying human disease, (i) epidemiological evidence associating a pathogen with a disease, (ii) presence of antibodies and/or immune cells against the specific antigens related with the disease, (iii) cross-reactivity of antibodies or immune cells of the host with microbial antigens and, (iv) reproduction by the antigen of the disease process *in vivo* or *in vitro* (Ang et al., 2004; Johnson et al., 2017).

Microbes can exhibit four types of molecular mimicry with the host proteins, (i) sequential or structural similarities with the proteins or protein-domains, (ii) similarities with the protein-structures without sequence homology, (iii) architectural similarities with the binding surfaces without sequence homology (interface mimicry) and, (iv) similarities with the short linear motifs (SLiMs) of the proteins (motif mimicry) (Xue et al., 2014; Dolan et al., 2015). SLiMs are short stretches of amino acids (3–10 amino acids) which are functionally diverse and mediate various signaling and protein-protein interactions (PPIs) (Davey et al., 2012; Dinkel et al., 2014; Garg et al., 2022). One another important functional component of a protein is molecular recognition features (MoRFs) 5–25 amino acid long. They are present in the structurally disordered region of protein hence disordered. But once MoRFs binds to the their partner, they attain a well-developed structure (Vacic et al., 2007; Yan et al., 2016). Reportedly, several viruses and bacteria propagate and sustain themselves inside the host by mimicking SLiMs of the host proteins (Sámano-Sánchez and Gibson, 2020).

PPIs and cell signaling play a pivotal role in the functioning of cellular life. Hence, mimicry of the host proteins involved in PPIs and cell signaling greatly helps microbes in modulating/disrupting the host defense mechanisms. Moreover, substitution of microbial proteins in the host PPI networks eventually shifts the paradigm of host-pathogen interactions in the favor of the pathogen. Several eukaryotic host-like SLiMs have been reported in viral proteins which helps in their entry inside the host cell and modulation of host cellular pathways related to transcription regulation, cell cycle, immune response etc. (Davey et al., 2011; Garamszegi et al., 2013; Hagai et al., 2014). Similarly, several eukaryotic host-like SLiMs have also been reported in bacterial proteins which helps them to interfere with signaling pathways involving protein tyrosine kinases (TKs) and mitogen-activated protein kinases (MAPKs), tyrosine phosphorylation etc., (Higashi et al., 2002; Frese et al., 2006; Li et al., 2007; Zhu et al., 2007; Sámano-Sánchez and Gibson, 2020). The emergence of antibiotic resistance in pathogens necessitates discovery of new anti-infective therapies. Recently, protein-based immune-modulatory molecules or drugs which can disrupt the host-pathogen PPIs have been proposed as a novel anti-infective therapy (Sámano-Sánchez and Gibson, 2020). Such therapeutics can interfere with the host-pathogen SLiM-mediated interactions and create a hostile and non-conducive host environment for the survival of the pathogen. Though, mimicry of the eukaryotic host-like SLiMs has been recognized as an important mechanism underlying microbial pathogenicity, presence and characteristics of host-like SLiMs have not been studied in the mimicry peptides (mimitopes) of bacteria and viruses experimentally associated with autoimmune diseases. Thus, the present study was conducted to discern if the experimentally verified microbial mimitopes underlying various autoimmune diseases exhibit motif mimicry with the host and potentially modulate the host PPIs. Additionally, the evolutionary pressure on microbial mimitopes was also determined. This is the first report on potential of autoimmunity-related microbial mimitopes in modulating host protein-protein interactions and their evolutionary characteristics.

## Materials and methods

### Retrieval of experimentally validated mimicry proteins from miPepBase

In the present study, the information on bacterial, viral and the host mimicry proteins was retrieved from a database of experimentally verified mimicry proteins, miPepBase (updated version assessed on January 2021). The database collates information about only the experimentally verified mimicry proteins/peptides and autoimmune diseases, thereof (Garg et al., 2017).

### Investigating the presence of eukaryotic host-like SLiMs in microbial mimicry proteins and mimitopes

The presence of eukaryotic host-like SLiMs in the microbial mimicry proteins and mimitopes was predicted using ANCHOR (Dosztányi et al., 2009). A eukaryotic host-like SLiM was considered to be present in the microbial mimitope if at least half of the amino acid of mimitopes overlapped with the SLiM region.

### Molecular evolutionary analyses of microbial mimitopes

The putative homologous/orthologous sequence of microbial mimicry proteins were identified using BLAST search against NCBI non-redundant (NR) protein database. To obtain a sequence homologous to each microbial protein, the NR database was searched using the microbial protein as query. Search results with query coverage of ≥80 and atleast 80% sequence identity were selected as the criteria for homology and used for further evolutionary analyses. The Orthologous protein clusters of each mimicry protein were aligned using ClustalW version 2 (Larkin et al., 2007). The corresponding gene sequences of these proteins were also aligned on the basis of their codons using pal2nal (Suyama et al., 2006). The ratio between the rate of non-synonymous substitution to the rate of synonymous substitution (*ω* = *d*N/*d*S) was used as a measure of the strength of selection pressure acting on a protein-coding gene. Assuming synonymous mutations are subjected to almost strictly neutral selection, the *ω* < 1, *ω* = 1, and *ω* > 1 represent negative selection, neutral evolution, and positive Darwinian selection, respectively (Yang, 2002). To compute the dN/dS ratio for each amino acid of the microbial mimitope, a site-specific model of the likelihood method was used using the codeml module of the PAML package (Yang, 2007).

## Result and discussion

Microbial mimicry of the host peptides has been implicated in several autoimmune diseases like multiple sclerosis (Wekerle and Hohlfeld, 2003), type 1 diabetes mellitus (Coppieters et al., 2012), autoimmune uveitis (Wildner and Diedrichs-Möhring, 2003), encephalomyelitis (Phelan et al., 2020), inflammatory bowel disease (Biank et al., 2007; Yusung and Braun, 2014), Crohn's disease (Cunha-Neto and Kalil, 2014; Polymeros et al., 2014), sarcoidosis, etc.

Motif mimicry of the eukaryotic host-like SLiMs has been associated with the pathogenicity of several bacteria and viruses because it helps them to hijack the host processes by rewiring the host PPI networks, signaling pathways, post-translational modifications etc., (Davey et al., 2012; Dinkel et al., 2014). Since, SLiMs are abundantly present in cell signaling and PPI network proteins, drugs targeting SLiM-regulated cellular processes have been proposed as novel therapeutics against bacterial and viral infections (Davey et al., 2011; Via et al., 2015; Sámano-Sánchez and Gibson, 2020). Thus, the present study was conducted to discern if the mimicry peptides (experimentally verified as responsible for autoimmune diseases; collated in miPepBase) contain the functional modules of PPIs, SLiMs and can be explored as novel drug targets.

A total of 150 bacterial mimicry proteins with their corresponding 28 host proteins and, 34 viral mimicry proteins with their corresponding 22 host mimicry proteins were retrieved from the miPepBase. The bacterial and their corresponding host mimicry proteins were named as Bacterial-set proteins. The viral and their corresponding host mimicry proteins were named as Viral-set proteins. The Bacterial-set proteins were involved in 16, while the Viral-set proteins were involved in 12 different types of autoimmune diseases. The detailed information of the host and pathogen, UniProtKB ID and name of the mimicry-protein(s), mimitope sequences, associated autoimmune diseases, and source of information (Pubmed ID) is provided in Supplementary Table 1.

During infection, the pathogens repurpose the host cells in a manner which is conducive to their own survival, proliferation and helpful for evading the host immunity. Thus, if the host cells can be rewired to the uninfected state or the one that is close to it, the disease progression can be halted or slowed down. Thus, drugs/molecules which can inhibit either (i) interactions between microbial host-like SLiMs and host PPI networks or, (ii) the metabolic chokepoints of these PPI networks or, (iii) nodes in the regulatory networks upstream or downstream of the hijacked host-SLiMs can be explored as novel therapeutics. Also termed as host-directed therapy, this approach has proved useful in combating many viruses and bacteria (Sámano-Sánchez and Gibson, 2020). The classical examples of host-directed therapy are the chemical inhibition of the proteasomal pathway which resulted in reduced viral loads in dengue infection and, RNA interference (RNAi) against some human proteins which inhibited replication of HIV and hepatitis C virus (Schwegmann and Brombacher, 2008). Our results revealed that the eukaryotic host-like SLiMs were abundant in the microbial mimicry proteins because 41 bacterial and 20 viral mimicry proteins showed the presence of SLiMs (Supplementary Table 2). On the contrary, only a few microbial mimitopes overlapped with the eukaryotic host-like SLiMs. Of the total 155 bacterial mimitopes, 10 bacterial mimitopes showed SLiMs and of the 43 viral mimitopes only 4 viral mimitopes had SLiMs (Table 1). This suggests that the most of the microbial mimitopes implicated in autoimmune diseases could not potentially rewire the host PPI networks for their own benefit. And, their pathology might have only the autoimmune component due to cross reactivity between the microbial epitopes and host peptides. However, the microbial mimitopes of ten bacteria and three viruses overlapped with the eukaryotic host-like SliMs (Table 1). Seven mimitopes, one each of *Agrobacterium tumefaciens, Yersinia enterocolitica, Escherichia coli, Propionibacterium freudenreichii, Streptococcus mutans, Lactobacillus johnsonii, Bifidobacterium longum* and two each of *Mycobacterium leprae* and Group A streptococcus showed the presence of eukaryotic host-like SLiMs in their mimitopes. Among the viruses, one mimitope of Herpes simplex virus and Herpes virus saimiri and two mimitopes of Epstein Barr virus had host-like SLiMs (Table 1). This suggests that these peptide regions might not only be responsible for inducing autoimmune diseases in the host by exhibiting epitope mimicry with the host, motif mimicry of the host-like SLiMs might also aid these bacteria and viruses in interrupting the host cell signaling and PPI networks.

**Table 1:** Detailed information about the microbial mimitopes which overlapped with the eukaryotic host-like SliMs.

**Sr. No**.	**Name and Uniprot id** **of the host mimicry** **protein (in** **parenthesis)**	**Autoimmune** **disease**	**Associated** **microbe**	**Name and Uniprot id** **of the microbial** **mimicry protein**	**Sequence of** **the microbial** mimitope*	**Position of the** **mimitope** **[Start-End]**	**Number of amino** **acids in the** **microbial** **mimitope**	**Number of amino** **acids in mimitope** **which show** **negative selection** **(*ω* < 1)**
1	Myelin basic protein of Mouse [P04370]	Encephalomyelitis	*Agrobacterium tumefaciens*	Replicating protein [P15394]	**AAQNRPSGPRK**	200–210	11	0
			*Yersinia enterocolitica*	YsaN [O30438]	**ANQTRPADIAA**	445–455	11	0
			*Propionibacterium freudenreichii*	Uroporphyrinogen III methyltransferase [Q51720]	**AHHVRPPALVV**	227–237	11	0
			Herpes simplex virus	Viral transcription factor ICP4 [P08392]	**AAQARPRPVAV**	790–800	11	0
			Herpes virus saimiri	Uncharacterized protein [Q80BM4]	**AAQRRPSRPFR**	125–135	11	11
2	Myelin basic protein of Cattle [P02687]	Leprosy	*Mycobacterium leprae*	50S ribosomal L2 [O32984]	**VSPWGKPEGRTRKPNKSSNK**	247–266	20	17
			*Mycobacterium leprae*	50S ribosomal L2 [O32984]	EQA**NINWGKAGRMRWKGKRP**	200–219	20	20
3	HEAE encephalitogen from myelin basic protein of rat [P02688]	Multiple Sclerosis	*Lactobacillus johnsonii*	FtsY [Q74IQ0]	**ESAEEVTTEDEQ**ER	125–138	14	5
4	Ro protein of human [P10155]	Systemic lupus erythematosus	Epstein Barr virus	Epstein_Barr virus nuclear antigen_1 (EBNA_1) [Q3KSS4]	**GGSGSGPRHRDGVRR**	58–72	15	0
5	Human skeletal myosin protein [P12883]	NA	*Escherichia coli*	Colicin protein [P02978]	**KAFQE**AEQR	148–156	9	8
6	SmD protein of human [P63162]	Systemic lupus erythematosus	Epstein Barr virus	Epstein_Ban nuclear antigen_1 (EBNA_1) [G0YSX7]	**PPPGRRP**	11–17	7	0
7	Human skeletal myosin protein [Q9UKX3]	Juvenile dermatomyositis	Group A streptococcus	M5 [P49054]	**ALEKLNK**EL	287–295	9	9
8	Myelin basic protein of rabbit [P25274]	Multiple Sclerosis	*Streptococcus mutans*	Permease [Q8DUP2]	**KTYGTLPSQD**	143–152	10	10
		Multiple Sclerosis	*Bifidobacterium longum*	Hypothetical [Q8G3X4]	**TNYGALPGSI**	9–18	10	10

* SLiM regions in the microbial mimitopes are shown in bold face.

Evaluation of the selection pressure on the microbial mimitopes revealed that 77.85% of the bacterial and 83.54% of the viral amino acids were under negative selection pressure (*ω* < 1), implying that microbial mimtopes were generally conserved or had a low mutation rate. Comas et al., reported that the T cell epitopes of *M. tuberculosis* were highly conserved which might be a distinct evolutionary strategy of a highly successful pathogen as *M. tuberculosis* for immune subversion (Comas et al., 2010). This suggests that other pathogens might also employ a similar strategy for a sustained survival inside the host. However, analysis of the microbial mimitopes which overlapped with the eukaryotic host-like SLiMs revealed that only 46.75% of the bacterial mimitopes and 25% of the viral mimitopes were under negative selection pressure (*ω* < 1) (Table 1; Supplementary Figure 1). This observation can be explained by the fact that SLiMs are in disordered regions of the proteins hence, the rate of mutation is high and these regions are usually under a positive selection pressure (Uyar et al., 2014). In past we have also shown that low complexity regions of proteins are not always disordered (Kumari et al., 2015).

Conventionally, autoimmune diseases like systemic lupus erythematosus, encephalomyelitis, multiple sclerosis etc., are treated using immunomodulators or immunosuppressants which only try to cure the symptoms but do not eliminate the etiological agent which continues to proliferate inside the host and interfering with vital cellular process(es). On the contrary, protein-based immune-modulatory molecules/drug molecules designed to inhibit the mimitopes (or eukaryotic host-like SLiMs) might also disrupt the host-pathogen PPIs. This implies that inhibitors of microbial mimitopes (or eukaryotic host-like SLiMs) identified in this study would not only help in treating the pathogen-associated autoimmune disease but also eliminate the etiopathological agent associated with the disease.

Finally, the repertoire of mimitopes discovered in this study suggests new paradigms underlying autoimmune diseases and the etiopathology associated with the infection. Such knowledge can provide important clues for the discovery of new drugs/protein-based immune-modulatory molecules against the pathogens.

## Author contributions

NS and MK conceived and designed the study. AG performed the work, prepared the illustrations, and wrote initial draft manuscript. All authors contributed to the critical analysis and revision, analyzed the results, and wrote the final versions of manuscript.

## Funding

The work was carried out using the resources funded by the Indian Council of Medical Research [Grant Nos: VIR(25)/2019/ECD-1 and ISRM/12(33)/2019]. AG was supported by ICMR-JRF scheme [Grant Number: 3/1/3 J.R.F.-2016/LS/HRD-(32262)]. NS was supported by CSIR Senior Research Associateship (Scientists' Pool Scheme) [Grant Number: 13(9089-A)/2019-Pool].

## Conflict of interest

The authors declare that the research was conducted in the absence of any commercial or financial relationships that could be construed as a potential conflict of interest.

## Publisher's note

All claims expressed in this article are solely those of the authors and do not necessarily represent those of their affiliated organizations, or those of the publisher, the editors and the reviewers. Any product that may be evaluated in this article, or claim that may be made by its manufacturer, is not guaranteed or endorsed by the publisher.
