# Supplementary figures and images for: Investigating the eukaryotic host-like SLiMs in microbial mimitopes and their potential as novel drug targets for treating autoimmune diseases
Source: Front Microbiol. 2022 Nov 4;13:1039188. doi: 10.3389/fmicb.2022.1039188 (PMC9672370; doi:10.3389/fmicb.2022.1039188)

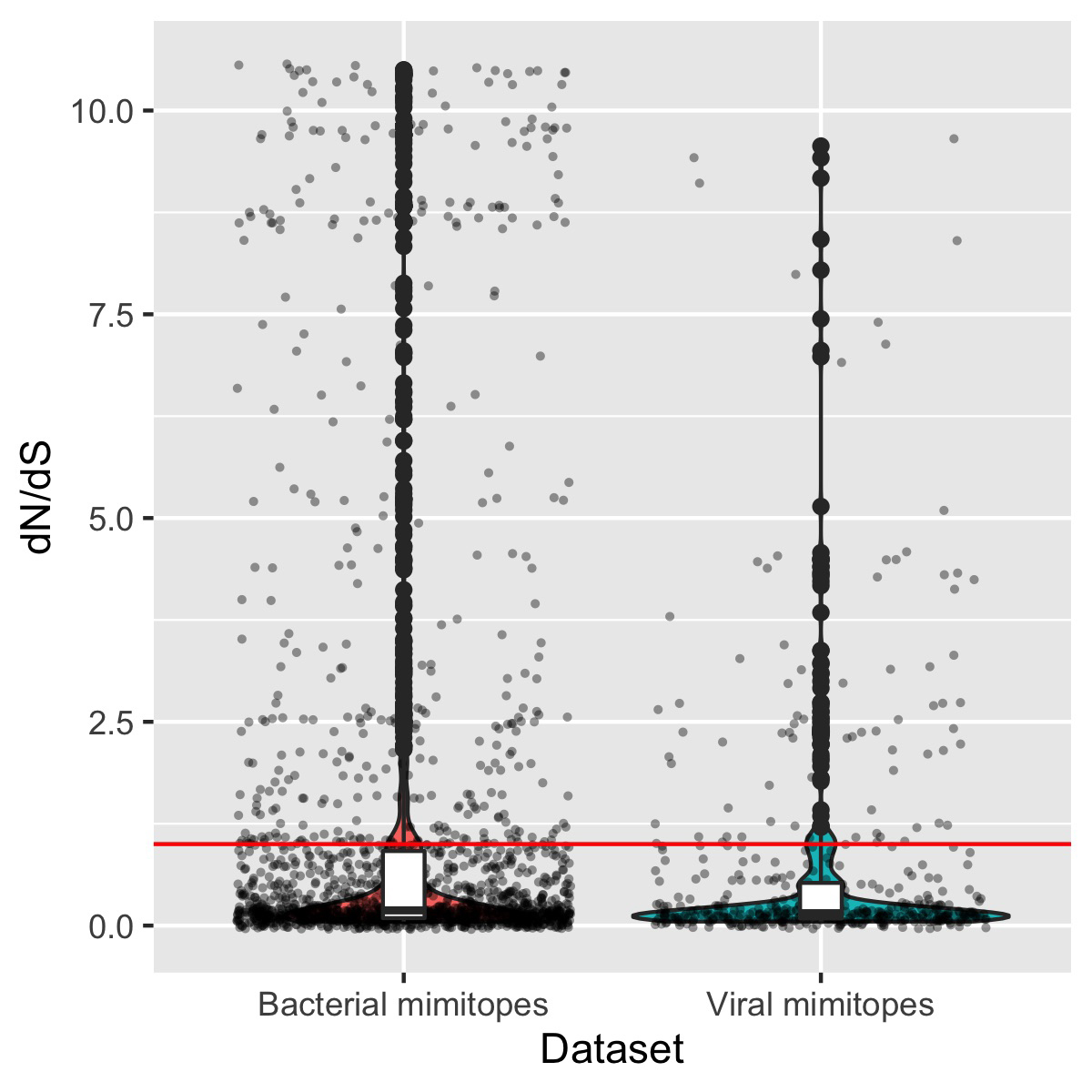

Supplement: Supplementary file 3 [file Image_1.JPEG]
